# Integration of mRNA and miRNA analysis reveals the molecular mechanisms of sugar beet (*Beta vulgaris* L.) response to salt stress

**DOI:** 10.1038/s41598-023-49641-w

**Published:** 2023-12-12

**Authors:** Ziqiang Zhang, Liang Wang, Wenjin Chen, Zengjuan Fu, Shangmin Zhao, Yuanyuan E, Hui Zhang, Bizhou Zhang, Mengyuan Sun, Pingan Han, Yue Chang, Kuangang Tang, Yanyan Gao, Huizhong Zhang, Xiaodong Li, Wenzhe Zheng

**Affiliations:** 1 Inner Mongolia Academy of Agricultural and Animal Husbandry Sciences, Hohhot, 010031 China; 2 Inner Mongolia Key Laboratory of Sugar Beet Genetics and Germplasm Enhancement, Inner Mongolia Academy of Agricultural and Animal Husbandry Sciences, Hohhot, 010031 China; 3 Linxi County Agriculture and Animal Husbandry Bureau, Chifeng, 025250 China

**Keywords:** Plant stress responses, Transcriptomics

## Abstract

The continuous increase of saline-alkali areas worldwide has led to the emergence of saline-alkali conditions, which are the primary abiotic stress or hindering the growth of plants. Beet is among the main sources of sugar, and its yield and sugar content are notably affected by saline-alkali stress. Despite sugar beet being known as a salt-tolerant crop, there are few studies on the mechanisms underlying its salt tolerance, and previous studies have mainly delineated the crop’s response to stress induced by NaCl. Recently, advancements in miRNA-mRNA network analysis have led to an increased understanding of how plants, including sugar beet, respond to stress. In this study, seedlings of beet variety "N98122" were grown in the laboratory using hydroponics culture and were exposed to salt stress at 40 days of growth. According to the phenotypic adaptation of the seedlings' leaves from a state of turgidity to wilting and then back to turgidity before and after exposure, 18 different time points were selected to collect samples for analysis. Subsequently, based on the data of real-time quantitative PCR (qRT-PCR) of salt-responsive genes, the samples collected at the 0, 2.5, 7.5, and 16 h time points were subjected to further analysis with experimental materials. Next, mRNA-seq data led to the identification of 8455 differentially expressed mRNAs (DEMs) under exposure to salt stress. In addition, miRNA-seq based investigation retrieved 3558 miRNAs under exposure to salt stress, encompassing 887 known miRNAs belonging to 783 families and 2,671 novel miRNAs. With the integrated analysis of miRNA-mRNA network, 57 miRNA-target gene pairs were obtained, consisting of 55 DEMIs and 57 DEMs. Afterwards, we determined the pivotal involvement of *aldh2b7*, *thic*, and *δ-oat* genes in the response of sugar beet to the effect of salt stress. Subsequently, we identified the miRNAs novel-m035-5p and novel-m0365-5p regulating the *aldh* gene and miRNA novel-m0979-3p regulating the *thic* gene. The findings of miRNA and mRNA expression were validated by qRT-PCR.

## Introduction

In order to cope with the increasing demand for food due to population growth, crop productivity needs to increase remarkably by 70–110% by 2050^1^. However, this calls for overcoming several challenges, with one of the primary challenges to achieving this goal is salt stress^2^, which severely impacts crop growth and development. More than 20% of irrigated land in the worlds affected by saline-alkali stress, resulting in reduced agricultural productivity^3^. In China alone, the saline soil area accounts for 1/3 of the total cultivated land area, which is estimated to be about 34 million ha^4^.

There has been extensive research on the osmotic regulation, physiological responses, and ion balance mechanisms of plants under NaCl stress, with numerous mechanisms of response being discussed relating to adaptation of plants to salt stress. Evidence exists reporting that an increase in osmotic adjustment compounds, including soluble sugars, alters the osmotic pressure under salt stress^5^. Furthermore, several other response mechanisms have also been widely explored, which includes the activated reactive oxygen species (ROS) scavenging activity to attenuate oxidative damage induced by rising salinity^6^ and the increased activity of Na^+^ and K^+^ transporters and channels to sustain the balance of cytosolic Na^+^/K^+^ ratio^7^.

Transcriptomics is a potent analytical method that enables the investigation of gene expression and function by measuring all the RNAs transcribed by a specific tissue or cell in a particular state^8^. Through this technique, crucial insights into the function and structure of target genes of interest can be obtained. On this basis, extensive research has been carried out on gene variable splicing^9^, transcript variation, and gene non-coding region function^10^. In addition, transcriptome sequencing approaches can detect not only genes that are expressed at low levels but also identify full-length transcripts without relying on the design of probes based on known sequences. This is particularly important in transcriptome studies of species where genomic information is relatively limited^11^. Transcriptome sequencing approaches have been widely applied in agriculture, including the exploration of resistance genes in animals and plants^12,13^, the study of resistance^14^, and metabolic mechanisms^15^.

Endogenous microRNAs (miRNAs) are short (18–24 nt) non-coding RNA molecules that negatively modulate gene expression after transcription and translation^16,17^. The miRNAs confer important roles in plant development and growth, including cell differentiation^18^, organ development such as the roots^19,20^, flowering^21,22^, and fruit development^23,24^. In addition to their physiological and metabolic roles, miRNAs are also actively involved in plants’ abiotic stress responses, including drought^25^, high temperature^26^, low temperature^27^, and saline-alkali stress^28,29^.

Beet is one of the widely utilized sources for global sugar production. In 2016–2017, sugar produced from beets reached 39.7 million tons, accounting for 22.5% of the total sugar output^30^. Beet exhibits a robust salt tolerant characteristic. Recent years have witnessed many studies on beet salt stress tolerance, focusing on different aspects such as antioxidant enzymes^31^, metabolites^32^, rhizosphere microorganisms^33^, transcriptome^34^, and miRNA regulation^35^. Our previous observations unveiled that the beet seedlings, under salt exposure, experienced initial wilting; however, this was later reversed later under salt stress. We, therefore, focused on deciphering the molecular mechanistic basis that underpins this response in beet seedlings by using the combination of RNA-seq and miRNA omics. Our study of adding miRNA was to supplement the regulatory mechanism of salt tolerance at the RNA level in seedlings.

## Results

### Determination of the time points used for sequencing

The mRNA expression patterns are presented in Fig. S1. According to the qRT-PCR results and the dynamic changes of "turgid–wilting–turgid" in sugar beet seedlings before and after salt stress, four time points, 0 (CK), 2.5 (S1), 7.5 (S2) and 16 h (S3) were selected for sequencing (Tables S1, S2).

### Evaluation of RNA sequencing data

To elucidate the molecular mechanisms underlying the phenotypic responses over time, we developed 12 mRNA libraries and 12 small RNA sequencing libraries using three biological replicates for both control and salt-stressed leaves, respectively. These libraries were sequenced on an Illumina HiSeqTM 2500 platform, yielded 13.31–16.89 million clean reads (Table 1) for four samples. Following data processing and filtering, 12.47–14.72 million clean tags were retained for further analysis (Table 1). The sequence reads were aligned to the beet genome sequence using Bowtie (Version 1.1.2), and the Match Ratio for all the samples exceeded 71.06%. The number of known and new miRNAs found in each samples summarized in Table 1.

**Table 1:** Summary of miRNA sequencing data.

Type	CK (0 h)	S1 (2.5 h)	S2 (7.5 h)	S3 (16 h)
Clean reads (× 10^6^)	14.42 ± 2.64	13.31 ± 7.76	16.89 ± 4.35	15.79 ± 7.26
Clean tags (× 10^6^)	13.35 ± 3.14	12.47 ± 7.23	14.72 ± 4.3	14.61 ± 7.33
Match ratio (%)	76.78 ± 9.09	74.91 ± 11.21	79.83 ± 3.59	71.06 ± 13.54
Known miRNA	265 ± 44	238 ± 79	276 ± 19	259 ± 38
Novel miRNA	1974 ± 70	1824 ± 584	1809 ± 313	2019 ± 328

For validation of the reliability of transcriptome data, qRT-PCR was conducted. According to the significant FPKM and TPM identified (Table S3), we selected 9 DEMs and 5 DEMIs associated with the salt stress response and evaluated their expression patterns at 0, 2.5, 7.5, and 16 h. The findings of qRT-PCR were basically consistent with the expression patterns in RNA-seq data (Tables S4, S5). Thus, DEMs expression was negatively correlated with DEMIs expression, thus confirming the accuracy of RNA-Seq method (Fig. S2). After salt stress, the expression level of miRNA in sugar beet seedlings is negatively correlated with the expression level of its target genes. Next, we plan to delve deeper into this relationship, hoping to reveal the intrinsic connections between miRNAs and target genes in sugar beet seedlings after salt stress.

Approximately 81.6 Gb of clean data were obtained by mRNA sequencing. The four samples yielded 42.54–50.71 million clean reads, and the Q30 base score was more than 92.80% in all of them. The clean reads were mapped to the *Beta vulgaris* L. reference genome by Tophat2 (2.1.1). The percentage of clean reads that were mapped to the reference genome ranged between 98.49 and 99.47%. The 88.60–89.94% uniquely mapped clean reads were used for the subsequent analyses (Table 2).

**Table 2:** mRNA sequencing dataset summary.

Type	CK (0 h)	S1 (2.5 h)	S2 (7.5 h)	S3 (16 h)
Clean data (bp) (× 10^9^)	6.65 ± 0.94	6.63 ± 0.82	6.35 ± 1.05	7.58 ± 4.30
Clean reads (× 10^6^)	44.52 ± 6.20	44.39 ± 5.48	42.54 ± 7.04	50.71 ± 28.79
% ≥ Q30 (%)	93.29 ± 0.74	92.80 ± 0.78	93.32 ± 0.82	93.23 ± 0.29
Mapped reads (%)	99.47 ± 0.30	98.49 ± 0.72	98.67 ± 0.24	99.08 ± 1.05
Unique match (%)	89.94 ± 0.81	89.31 ± 1.09	88.60 ± 1.62	89.29 ± 1.81

### DEMIs and DEMs

**Figure 1:**
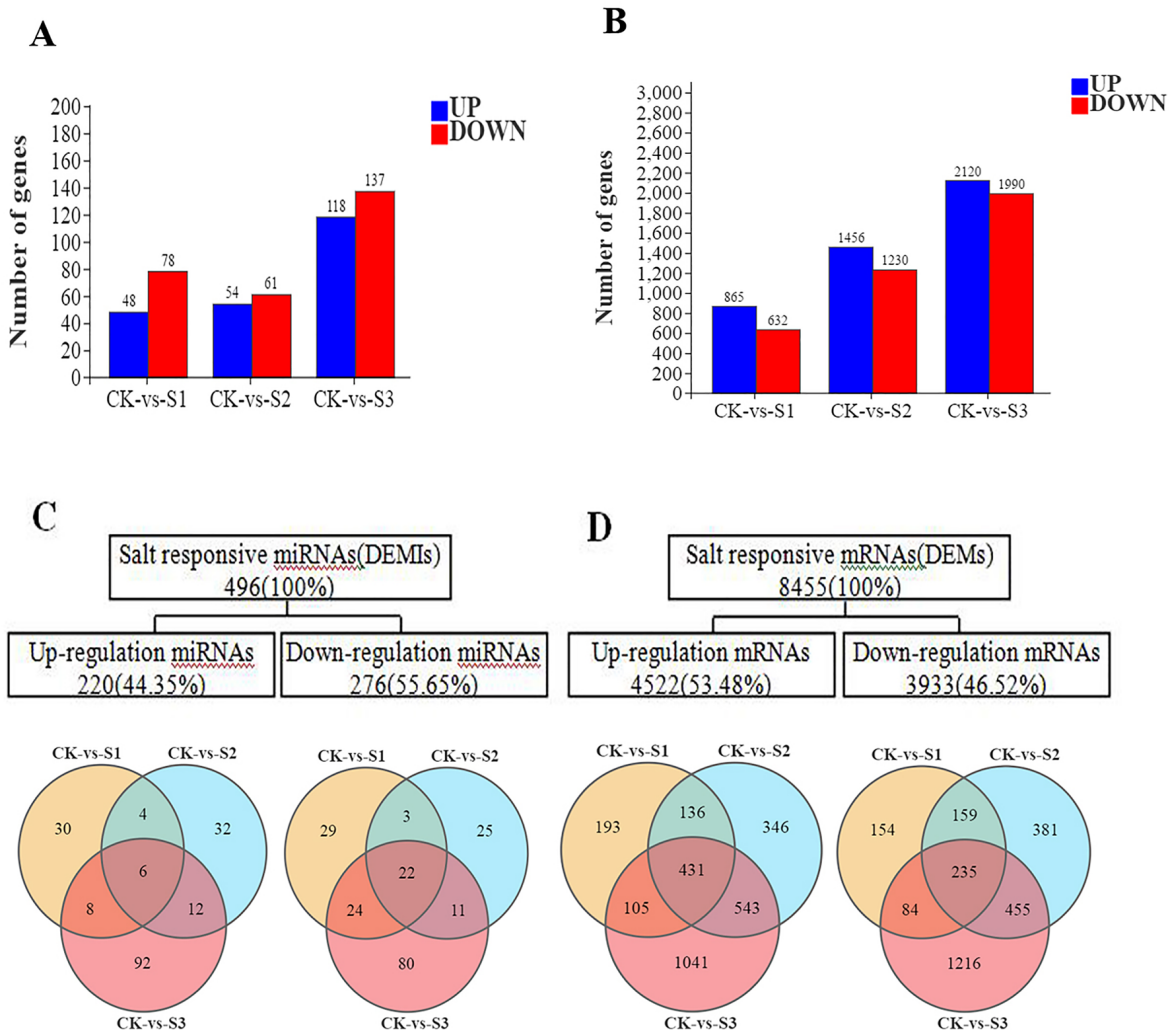
The expression of salt-stress-regulated DEMIs and DEMs in sugar beet seedlings. (**A**, **B**) Column diagrams representing the DEMI and DEM numbers. (**C**, **D**) Venn diagrams demonstrating the DEMI and DEM numbers and the set overlaps obtained across three comparisons.

The number of DEMIs and DEMs in the samples gradually increased along with the sampling time after stress application (Fig. 1A,B). In total, 496 DEMIs were discovered, with 44.35% and 55.65% up- and down -regulated DEMIs, respectively. Moreover, 8455 DEMs were detected, of which 53.48% and 46.52% were up-and down-regulated, respectively (Fig. 1C,D). The raw data for mRNA and miRNA sequencing in Fig. 1 are shown in Tables S6 and S7.

### Functional analysis of DEMs

In order to clarify the impact of differential expression patterns on DEM functions, we categorized 8455 DEMs as per their expression trends, resulting in the establishment of 20 distinct trends. The top five trends, which encompassed 4969 DEMs (Fig. S3A), accounted for more than 58.77% of the total.

After salt stress treatment, we conducted trend analysis on differentially expressed genes, and in the results, we used "profile" to represent different trend modules based on differentially expressed gene clustering. According to the dynamic changes of "turgid–wilting–turgid" phenotypes before and after salt stress, combined with the trend of gene expression, we focused on the following five profiles: continuously decreasing (profile 0), invariable–decreasing (profile9), invariable–increasing (profile 10), invariable–increased–decreasing (profile 12), continuously increasing (profile 19). 881 DEMs were down-regulated in profile 0. As per the GO results, these DEMs were primarily enriched in "cellular process (GO: 0009987)", "metabolic process (GO: 0008152)", and "catalytic activity (GO: 0003824)".

During the early stages of salt stress, 1447 DEMs in profile 9 showed no significant alterations in the expression; however, they began to decrease 7.5 h post-NaCl administration. GO analysis revealed that these DEMs were predominant in "metabolic process (GO: 0008152)", "cellular process (GO: 0009987)", "cell (GO: 0005623)", and "cell part (GO: 0044464)".

In contrast with the above, 961 DEMs in Profile 10 underwent no remarkable changes in the early stages of salt stress; however, they started to increase 7.5 h post-NaCl administration. These DEMs were mainly enriched in the GO categories "catalytic activity (GO: 0003824)", "metabolic process (GO: 0008152), and "membrane (GO: 0016020)".

The expression of 586 DEMs in Profile 12 did not change evidently in the early stages of salt stress. They increased early, 2.5 h after NaCl application; however, they reverted to their control expression after 7.5 h. These DEMs were primarily enriched in the GO categories "catalytic activity (GO: 0003824)", "metabolic process (GO: 0008152)", and "cellular process (GO: 0009987)".

The expression pattern of 1094 DEMs in Profile 19 exhibited, after an initial up-regulation in the early stage of salinity stress, a stable trend throughout the later stages of stress conditions. These DEMs were primarily enriched in the GO categories "catalytic activity (GO: 0003824)", "single-organism process (GO: 0044699), and "response to stimulus (GO: 0050896)". Significant differences were noted in the GO categories enriched among the DEMs exhibiting different expression trends, particularly with regards to those that were subjected to up-regulation and down-regulation (Fig. S3B).

Functional differences and similarities between DEGs after salt stress were further explored by KEGG pathway enrichment analysis of 4522 up-regulated and 3933 down-regulated mRNAs, respectively, and identified 20 pathways with the highest expression, enrichment levels (Fig. S4). Out of these, five pathways were shared by up- and down-regulated DEMs, namely "biosynthesis of secondary metabolites (ko01110)", "starch and sucrose metabolism (ko00500)", "plant hormone signal transduction (ko04075)", "cysteine and methionine metabolism (ko00270)", and "metabolic pathways (ko01100)". Permission has been obtained from Kanehisa laboratories for using KEGG pathway database^36^.

### Development of a network of co-expression between DEMIs and DEMs

**Figure 2:**
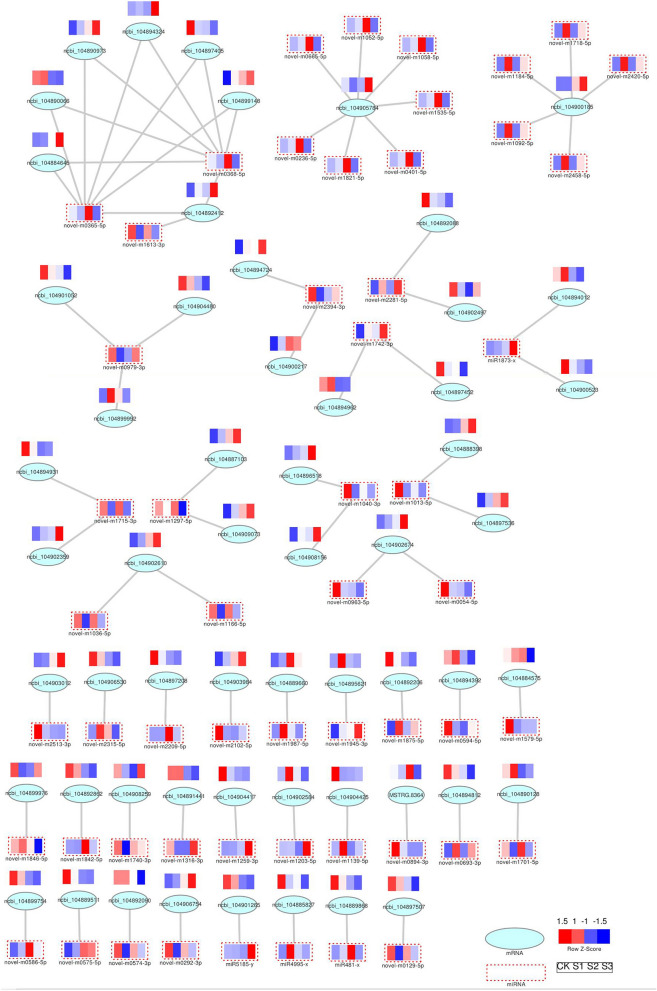
Under salt stress, the miRNA-mRNA correlation network in sugar beet seedlings.

**Figure 3:**
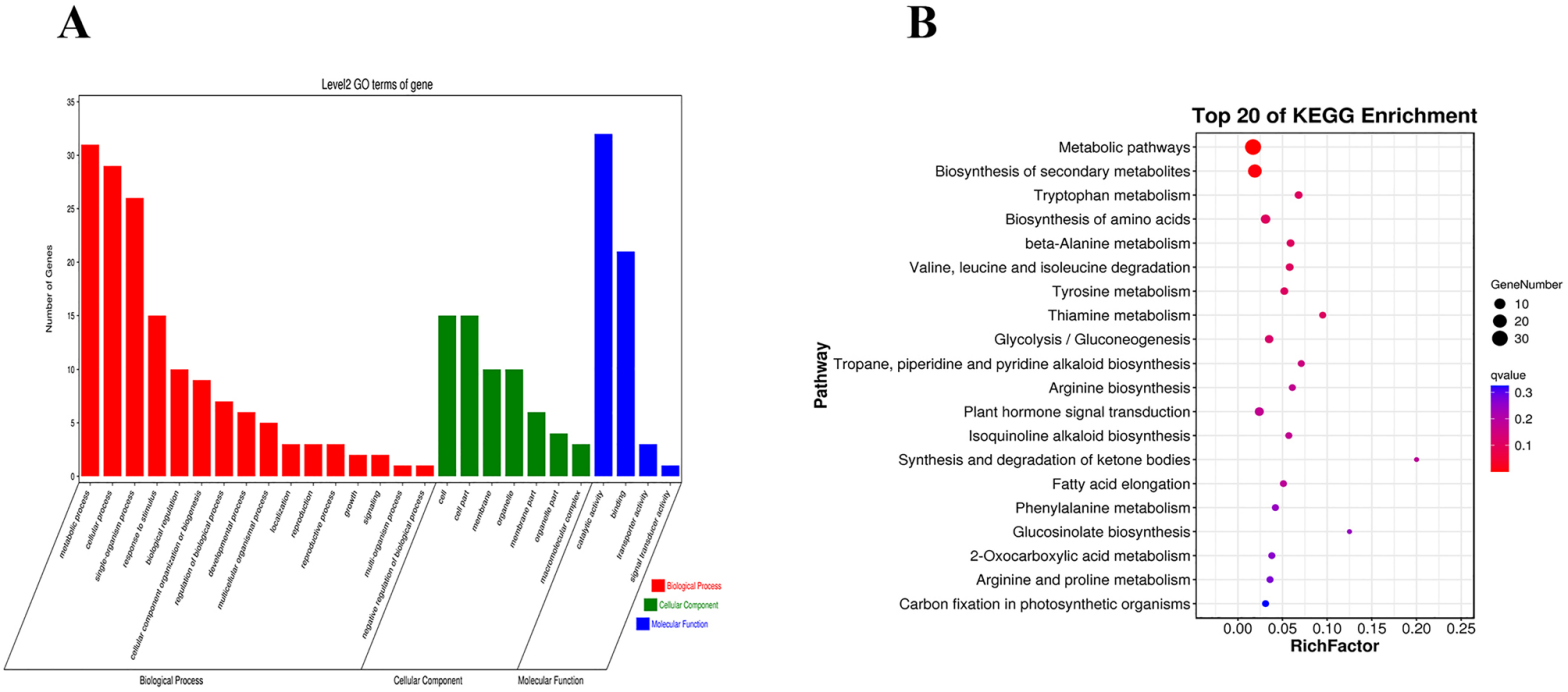
GO and KEGG enrichment analysis of coexpression network associated genes between DEMIs and DEMs. (**A**) GO enrichment analysis results, (**B**) KEGG enrichment analysis results.

Under salt stress, a regulatory network containing DEMIs and DEMs was constructed to examine the link between miRNAs and mRNAs (Fig. 2). GO enrichment analysis revealed that 55 DEMIs negatively modulated the expression of 57 DEMs. Moreover, the target genes enriched in 27 GO terms of molecular function (MF), biological process (BP), and cellular component (CC). Among them, a higher number of genes were enriched in the "catalytic activity (GO: 0003824)", "metabolic process (GO: 0008152),"and "cellular process (GO: 0009987)" categories (Fig. 3A). As per the KEGG analysis results, the target genes were enriched in 60 pathways, such as "biosynthesis of secondary metabolites (ko01100)", "metabolic pathways (ko01100)", and "biosynthesis of amino acids (ko01230)" (Fig. 3B).

### Analysis of key genes involved in sugar beet response to salt stress

In our work, we focused on three genes, aldehyde dehydrogenase family 2 member B7 (*aldh2b7*), phosphomethylpyrimidine synthase (*thic*), and ornithine aminotransferase (*δ-oat*), based on their significant expression changes before and after salt stress treatment. Relative to the control, the expression of *aldh2b7* was subjected to up-regulation at all timepoints after salt stress. Similarly, *δ-oat* gene expression was up-regulated continuously. On the other hand, *thic* gene expression was up-regulated at the initial stages of salt stress but experienced a down-regulation at 16 h.

## Discussion

At present, studies on molecular regulatory mechanisms related to salt tolerance in plants mainly focus on mRNA, but biological gene regulatory mechanisms are abundant, including the regulation of mRNA by non-coding RNA (such as miRNA), mRNA targeted by miRNA, and mRNA targeted by miRNA. It can inhibit, cut and even degrade the mRNA it targets, thus affecting the function of the mRNA in the organism. In the process of salt stress treatment of beet seedlings, we found that seedlings with strong salt tolerance would show a dynamic change of "turgid–wilting–turgid" before and after stress. In order to analyze the molecular regulatory mechanism behind this biological phenomenon, we first cultured beet seedlings with nutrient solution, and exposed to salt stress at the third true leaf growth stage. Plant samples were harvested at specific intervals, and using the qPCR method, we determined the optimal sequencing time points as being 0, 2.5, 7.5 and 16 h. Subsequently, we conducted an integrated analysis of miRNA and mRNA expression patterns in roots to examine the putative mechanisms involved in the salt stress response. Our data uncovered useful miRNA–mRNA gene pairs that respond to salt stress by constructing a modulatory network between DEMIs and DEMs. The current exploration elucidated the interactions and potential roles in response to salt stress in sugar beets. Besides, we evaluated the expression changes pre- and post-stress application and between treatments to illustrate the involvement of specific genes in salt stress responses. We hope that these findings can supplement the RNA molecular regulatory mechanism of salt tolerance in sugar beet.

### miRNA sequencing analysis

miRNAs, which are crucial non-coding modulatory small RNAs, confer crucial roles in gene modulation of a majority of eukaryotes as well as in abiotic and biotic stress responses^37–39^. Thus, it is of importance to evaluate miRNA expression and their regulatory roles in sugar beet during salt stress. The subsequent miRNA-seq analysis data unveiled 3558 miRNAs in response to salt stress, comprising 887 known miRNAs from 783 families, as well as 2671 novel miRNAs. The identified sequences were subjected to comparison with those present in the miRBase database^40^. Among them, 887 known miRNAs were recognized, belonging to 783 different miRNA families (Tables S8, S9). Of note, prior research has documented that certain miRNA families, including miR-156^41,42^, miR-167^43^, miR-319^44^, miR-396^45,46^, miR-398^42,47^, and miR-399^48^ were functionally significant in response to saline-alkali or drought stress in other plants.

Among these sequences, we found 22 miRNAs (Table S10) that were negatively correlated with *aldh*, *thic*, and *δ-oat* target mRNAs. Finally, we obtained 3 miRNAs linked to beet response to salt stress. These were novel-m0365-5p, novel-m0368-5p and novel-m0979-3p. Novel-m035-5p and novel-m0365-5p regulate *aldh*, and novel-m0979-3p regulates *thic*. Further research into their functions and involvement under abiotic stress in plants would be intriguing.

### mRNA sequencing analysis

By performing an mRNA dynamic expression analysis of sugar beet under salt stress, we identified 8455 DEMs at 0, 2.5, 7.5 and 16 h, which included 4522 and 3933 up- and down-regulated genes, respectively. Notably, KEGG analysis unmasked an enrichment of several pathways that commonly occurred in both up- and down-regulated genes, such as "plant hormone signal transduction", "biosynthesis of secondary metabolites", "cysteine and methionine metabolism", and "starch and sucrose metabolism". Considering the pre- and post-stress expression changes, together with the differences between treatments, we highlighted three genes of interest: *aldh2b7*, *thic*, and *δ-oat*. These three genes have been previously identified to be responsive to salt stress across a range of salt experiments, thus enhances the reliability of our results. In *V. fordii*, *aldh2b7* gene expression was found to be related to abiotic stress like high salt, drought, and abscisic acid^49^. Another investigation confirmed a significant up-regulation of the thic gene expression in the early stages of salt stress^50^. Collectively, these studies support the notion that *aldh2b7*, *thic*, and *δ-oat* are key genes involved in sugar beet responses to salt stress.

### Integrated analysis of DEMIs and DEMs

Using miRNA-mRNA integration analysis, recent investigations have broadened our knowledge of how plants respond to stress^14,39,42,51^. This current study identified several miRNA-target gene pairs that respond to salt stress and developed a regulatory network using DEMIs and DEMs. Mounting studies have highlighted the association of plant hormone signal transduction with certain miRNA target genes^52,53^. Interestingly, certain differentially expressed target genes are inversely regulated by miRNAs, such as miR-5185-y, miR-4995-x, and miR-481-x. It is possible to understand the role miRNAs play in plant salt stress responses based on the differential expression of the target genes after stress.

### The role of *aldh2b7* gene

Extensive research indicates that *aldh* is a gene family with great importance in plant stress resistance by participating in various plant metabolic processes linked to abiotic stress adaptation. *Aldh* is an important enzyme in the detoxification of ROS. Specifically, it can catalyze the oxidation of toxic aldehydes, converting them into non-toxic carboxylic acids, thereby maintaining the microbalance of aldehydes in plants^54–56^. The *aldh* proteins act to mitigate the damage of abiotic stress by catalyzing the irreversible conversion of aldehydes to acids. In contrast to wild-type Arabidopsis thaliana, transgenic Arabidopsis plants over expressing *aldh7b-15a* gene have displayed enhanced tolerance to drought-induced stress, as evidenced by the regulation of stress-responsive genes, maintenance of root growth, retention of water and chlorophyll content and a reduction in MDA content^57^.

### The role of *thic* gene

Thiamine plays an important role in plant growth and development, as well as in response to biotic and abiotic stresses. Thiamine is a bicyclic compound connected to thiazole and pyrimidine. In the biosynthesis process of thiamine, pyrimidine and thiazole are synthesized separately, and ultimately coupled to form thiamine pyrophosphate form. Pyrimidine synthase (THIC), thiazole synthase (THI1), thiamine phosphate synthase (TH1), and thiamine pyrophosphate kinase (TPK) are key enzymes in the thiamine synthesis pathway, regulating the synthesis of thiamine in plants^58^. The biosynthesis of pyrimidine part is to synthesize 4-amino-2-methyl-5-hydroxymethyl pyrimidine monophosphate (4-amino-2-methyl-5-hydro) with 5-amino imidazole ribonucleoside (Air) as the substrate and *thic* as the catalyst. This process needs S-adenosylmethionine (Sam) and reducing amide NADH as cofactors. Under the catalysis of thiamine phosphate synthase, HMP-P forms into 4-amino-2-methyl-5-hydroxymethylpyrimidine pyrophosphate (HMP-PP)^59^.

### The role of *δ-oat* gene

Proline is related to plant stress resistance. Research has shown that proline activity increases with increasing salt concentration and treatment time. Under normal growth conditions, *δ-oat* is mainly involved in the mobility of nitrogen required for plant growth. However, the highly significant positive correlation between proline and *δ-oat* activity under salts tress conditions suggests that everything path contributed to proline synthesis^60^. In higher plants, two pathways of proline biosynthesis have been demonstrated, one from glutamate, and the other from ornithine^61,62^. The role of the glutamate pathway in proline accumulation is well established and has been shown to be the predominant pathway in response to osmotic stress^61^. Concerning ornithine pathway, *δ-oat* has been shown to participate in proline synthesis. It catalyzes the loss of the ornithine-d-amino group from ornithine to provide pyrroline-5-carboxylate (P5C) in mitochondria. The product formed is transported into the cytoplasm which is further reduced to proline by P5CR^63^.

In this research, it was found that when subjected to salt stress, sugar beet (*Beta vulgaris* L.) exhibits phenotypic adaptation of the seedling leaves, transitioning from turgidity to wilting and then back to turgidity before and after treatment. The *aldh2b7*, *thic*, and *δ-oat* genes were identified as being crucial for sugar beet response to salt stress. Subsequent investigations clarified the miRNAs novel-m035-5p and novel-m0365-5p as miRNAs responsible for regulating the *aldh* gene and miRNA novel-m0979-3p as a miRNA responsible for regulating the *thic* gene. By exploring the molecular mechanisms underlying sugar beet’s salt stress response at the miRNA level, this study casts new light on the potential molecular pathways and identifies new opportunities for breeding sugar beets with facilitated salt stress tolerance.

## Materials and methods

### Hydroponic culture

We acknowledge the use of plant materials in this manuscript complies with all relevant institutional, national, and international guidelines and legislation. The beet cultivar "N98122" specimen in this study was from the Special Crops Institute of Inner Mongolia Academy of Agricultural and Animal Husbandry Sciences. To carry out the experiment, we utilized hydroponics culture, which was conducted in a culture chamber with artificial light. Uniformly sized beet seeds were selected, sown in a beet germinating box, and placed in an incubator maintained at a constant temperature of 25 °C in the dark to enable germination. After emergence, the seedlings were placed under light, and beet seedlings with uniform size were selected and transplanted into 50-L culture tanks with 200 holes in each tank, with one plant placed per hole. The hydroponics culture was maintained using modified Hoagland nutrient solution renewed every 7 days. The illumination intensity was (450 ± 50) μmol m^−2^ s^−1^), with a 14 h light period daily, the daytime temperature was set at (25 ± 1) °C, the night temperature was set at (20 ± 1) °C, and the relative humidity was 60–70%.

### Sample collection

When the fourth true leaf of a beet seedling grows to approximately 2.7 cm × 1.0 cm, salt stress was administered by introducing NaCl into the nutrient solution, resulting a final NaCl concentration of 300 mM. Three seedling leaves were randomly sampled before salt treatment to establish a control setup CK (0 h). Based on the wilting response of beet seedlings, the following 17 sampling time points were set: 0.5, 1, 1.5, 2, 2.5, 3, 3.5, 5.5, 7.5, 12.5, 13.5, 14, 14.5, 15, 15.5, 16, and 24.5 h. Subsequently, samples were collected at each time point, whereby the seedlings were quickly frozen using liquid nitrogen for 1 h, and then stored in a − 80 °C freezer for preservation. All the materials are taken from the third true leaf of the beet seedling, at each time point; three replicates were set, resulting in 51 samples in total, including controls.

### RNA extraction, library construction and sequencing

Total RNA was extracted using Trizol reagent kit (Invitrogen, Carlsbad, CA, USA) according to the manufacturer’s protocol. RNA quality was assessed on an Agilent 2100 Bioanalyzer (Agilent Technologies, Palo Alto, CA, USA) and checked using RNase free agarose gel electrophoresis. After total RNA was extracted, eukaryotic mRNA was enriched by Oligo (dT) beads, while prokaryotic mRNA was enriched by removing rRNA by Ribo-Zero TM Magnetic Kit (Epicentre, Madison, WI, USA). Then the enriched mRNA was fragmented into short fragments using fragmentation buffer and reverse transcripted into cDNA with random primers. Second-strand cDNA were synthesized by DNA polymerase I, RNase H, dNTP and buffer. Then the cDNA fragments were purified with QiaQuick PCR extraction kit (Qiagen, Venlo, The Netherlands), end repaired, poly (A) added, and ligated to Illumina sequencing adapters. The ligation products were size selected by agarose gel electrophoresis, PCR amplified, and sequenced using Illumina HiSeq™ 2500 by Gene Denovo Biotechnology Co. (Guangzhou, China).

### Small RNA sequencing and analysis

After total RNA was extracted by Trizol reagent kit (Invitrogen, Carlsbad, CA, USA), the RNA molecules in a size range of 18–30 nt were enriched by polyacrylamide gel electrophoresis (PAGE). Then the 3ʹ adapters were added and the 36–44 nt RNAs were enriched. The 5ʹ adapters were then ligated to the RNAs as well. The ligation products were reverse transcribed by PCR amplification and the 140–160 bp size PCR products were enriched to generate a cDNA library and sequenced using Illumina HiSeq™ 2500 by Gene Denovo Biotechnology Co. (Guangzhou, China). For identification and removal of rRNAs, scRNAs, sonRNAs, snRNAs, and tRNAs, only those clean tags received alignment with the small RNAs in the GeneBank (Release 209.0) and Rfam databases (11.0). Using the reference genome, the clean tags were aligned and any reads corresponding to repeat sequences, exons, or introns were eliminated. The miRBase database (Release 22) was searched to identify known sugar beet miRNAs among all clean tags. A reference genome alignment was performed on all unannotated tags. The program Mireap_v0.2 allows the identification of novel miRNA candidates as per their genomic locations and hairpin structures.

### Enrichment analysis of miRNAs

The target gene candidates differentially expressed miRNAs (DEMIs) (referred to "target gene candidates" hereinafter) were subjected to GO analysis, which was conducted according to the GOseq-based Wallenius non-central hyper-geometric distribution.

The p-value was subjected to correction by FDR method and FDR ≤ 0.05 was a threshold. Pathways that meet this condition were considered significantly enriched ones in DEMIs. Functional enrichment of both target genes of miRNAs were carried out in single samples and DEMIs in a compare group.

### mRNA sequencing and analysis

Oligo (dT) beads were involved in the enrichment of the mRNA after total RNA extraction. To construct the library, cDNA underwent reverse transcription utilizing random short sequence primers as templates. QiaQuick PCR extraction kit was applied to purify the cDNA fragments by applying DNA polymerase I, dNTP, RNase H, and buffer. A Poly (A) tail was added and Illumina sequencing adapter ligation to the Illumina HiSeqTM 2500 was applied by Gene Denovo Biotechnology. Then paired-end reads were synthesized. To acquire clean reads, we eliminated low-quality reads, adapter-containing reads, and ploy-N reads. Next, the clean reads were mapped to sugar beet related reference genome sequence (RefBeet-1.2.2) (https://www.ncbi.nlm.nih.gov/datasets/genome/GCF_000511025.2/). The drawing software pheatmap (https://cran.r-project.org/web/packages/pheatmap/index.html) was used to draw heatmaps of gene expression levels.

### Developing a DEMI-targeted (DEM) network

Using the web tool IDEG6, fragments per kilobase per million reads (FPKM) values were first compared across the samples to identify DEMs^64^. A miRNA was considered a DEM if it had an FDR < 0.01 and |log2 Fold Change (FC)| ≥  2, between treatments, based on three biological replicates. Likewise, a miRNA with an FDR < 0.05 and |log2FC| ≥ 1 was defined as a DEMI. Patmatch (v1.2) software analysis enabled the identification of the target genes of DEMIs obtained from DEMs. Subsequent to obtaining the negatively correlated DEMI-target gene pairs, visualization was done using Adobe Illustrator CS6 (San Jose).

### Functional analysis of DEMs

KEGG (http://www.genome.jp/kegg/) and GO (http://www.geneontology.org/) databases were used to perform mRNA and miRNA functional enrichment analysis. Cluster Profiler was utilized for pathway enrichment analysis of DEG sets on the basis of the hypergeometric distribution principle.

### qRT-PCR

In order to select the appropriate time points for sequencing, qRT-PCR was conducted on the 18 time points that were sampled. We selected three genes related to salt tolerance, NHX1^65^, NHX6^66^, and VSHE8^67^, and detected their changes in the expression relative to reference genes at varied time points. To validate the transcriptome sequencing data, *gadph* served as the loading control for mRNA expression analysis and *U6* snRNA for miRNA expression analysis, respectively. Five DEMIs and nine DEMs were selected for qRT-PCR validation. The qRT-PCR primers were designed utilizing the reference gene sequence using Primer 5.0 (Table S11).

MiRNAs and mRNAs were produced by Sangon Biotech Co., Ltd. using the Mir-X miRNA First-Strand Synthesis [iScript gDNA Clear cDNA Synthesis Kit (Biorad, America)]. On the fluorescence quantitative PCR system, TB Green Premix Ex Taq II kit (Takara, Dalian, China) was adopted for quantitative analysis. Each sample was run in three technical replicates, and relative expression of DEMIs and DEMs was computed by 2^−ΔΔCt^ method^68^.

### Supplementary Information

Supplementary Information.

## Data Availability

The raw sequence data reported in this paper have been deposited in the Genome Sequence Archive (Genomics, Proteomics & Bioinformatics 2021) in National Genomics Data Center (Nucleic Acids Res 2022), China National Center for Bioinformation / Beijing Institute of Genomics, Chinese Academy of Sciences (GSA: CRA010166) that are publicly accessible at https://ngdc.cncb.ac.cn/gsa.
