# Supplementary material for: Integration of mRNA and miRNA analysis reveals the molecular mechanisms of sugar beet (Beta vulgaris L.) response to salt stress
Source: Sci Rep. 2023 Dec 12;13:22074. doi: 10.1038/s41598-023-49641-w (PMC10716384; doi:10.1038/s41598-023-49641-w)
Supplement: Supplementary file 1 — Supplementary Information. [file 41598_2023_49641_MOESM1_ESM.zip › Figture S3.pdf]

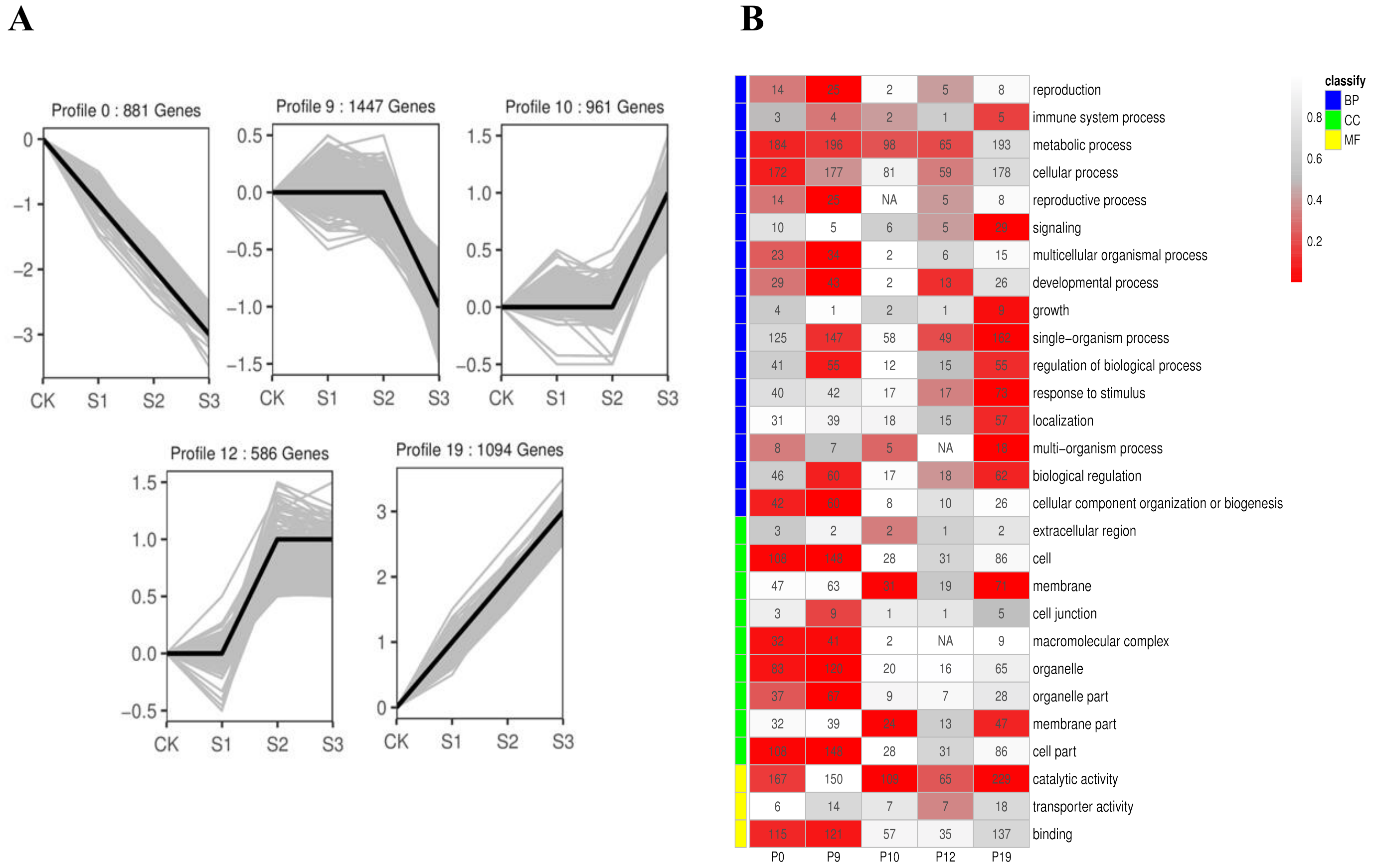

Fig. S3. GO enrichment analysis across four time points during salt stress of sugar beet seedling stage. (A) Four representative profiles of different expression trends. In each frame, the light gray lines represent the expression pattern of individual genes, while the black line represents the expression tendency of all the genes. (B) GO enrichment analysis of four significant clusters of genes in salt stress. (The smaller the p-value, the redder the color; Pathways (NA) not enriched, filled in white. The number in the grid represents the number of genes enriched in the trend module onto the GO term).
