# Supplementary material for: Integration of mRNA and miRNA analysis reveals the molecular mechanisms of sugar beet (Beta vulgaris L.) response to salt stress
Source: Sci Rep. 2023 Dec 12;13:22074. doi: 10.1038/s41598-023-49641-w (PMC10716384; doi:10.1038/s41598-023-49641-w)
Supplement: Supplementary file 1 — Supplementary Information. [file 41598_2023_49641_MOESM1_ESM.zip › Figture S4.pdf]

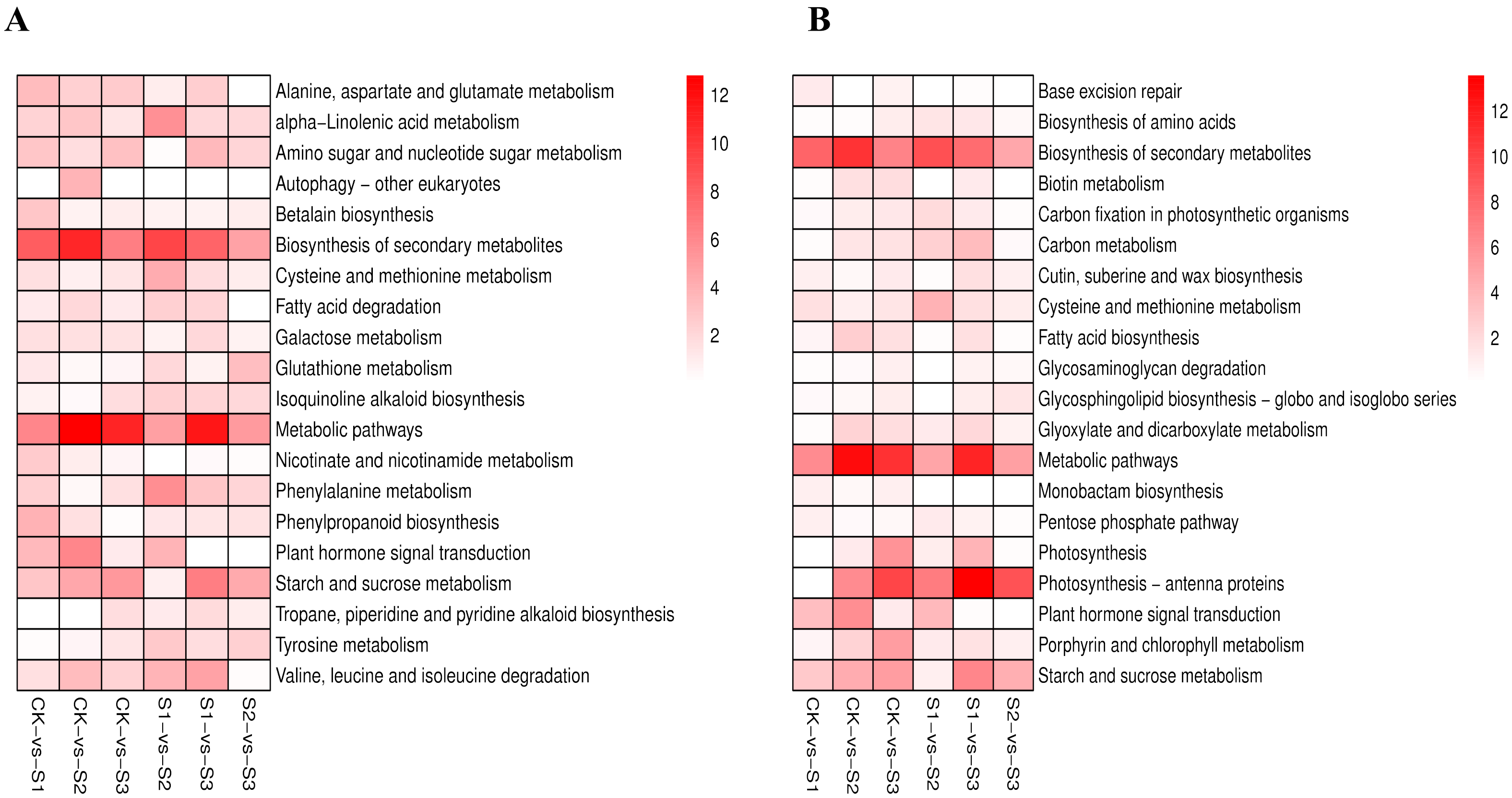

Fig. S4. KEGG enrichment analysis across four time points during salt stress of sugar beet seedling stage. (A) KEGG enrichment analysis of up- regulated DEGs. (B) KEGG enrichment analysis of down- regulated DEGs. (The smaller p-value, and the redder the color).
