# Supplementary figures and images for: Integration of mRNA and miRNA analysis reveals the molecular mechanisms of sugar beet (Beta vulgaris L.) response to salt stress
Source: Sci Rep. 2023 Dec 12;13:22074. doi: 10.1038/s41598-023-49641-w (PMC10716384; doi:10.1038/s41598-023-49641-w)

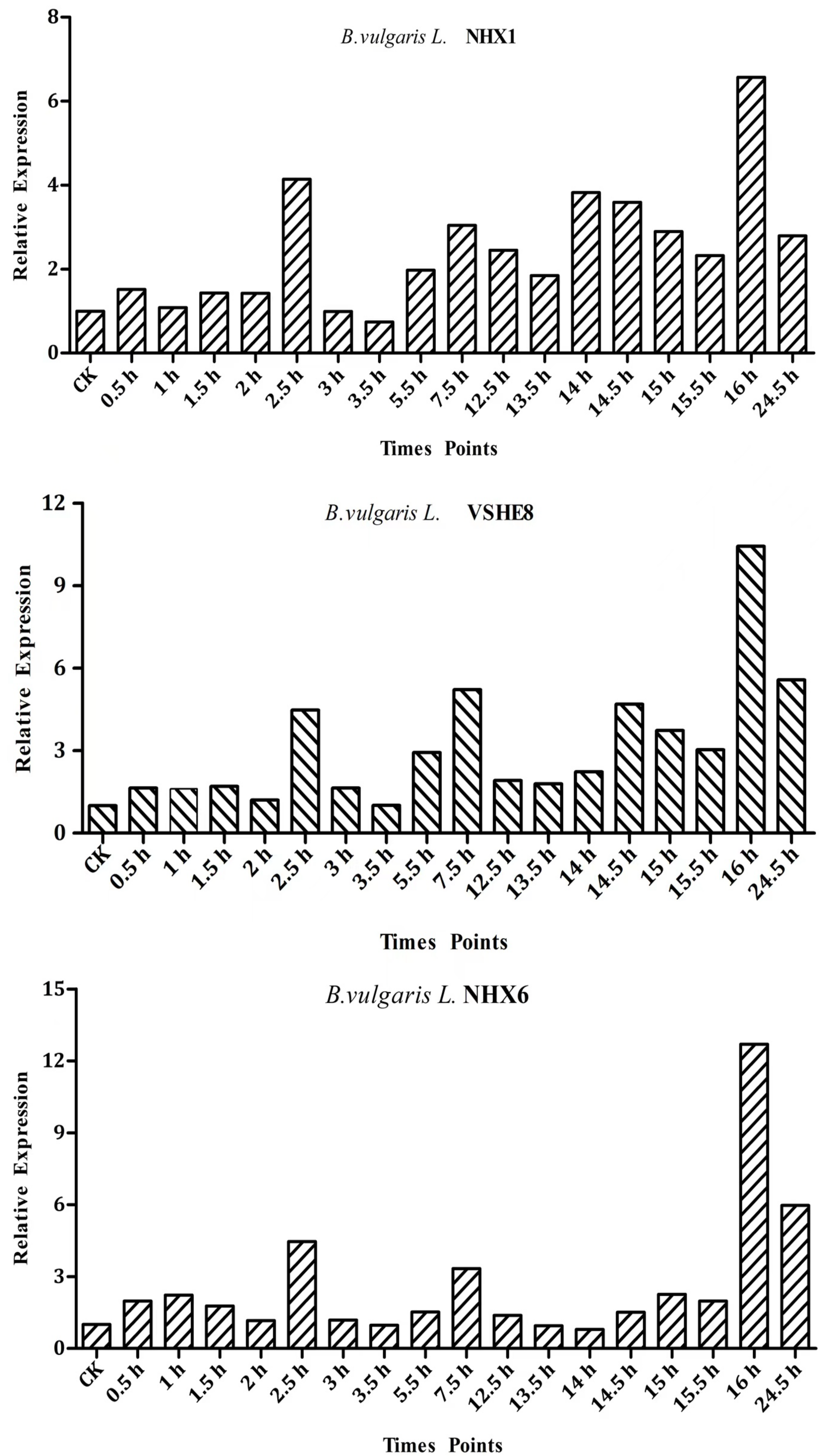

Fig. S1. Detection of mRNA expression levels at different time points using qRT-PCR.

Supplement: Supplementary file 1 — Supplementary Information. [file 41598_2023_49641_MOESM1_ESM.zip › Figture S1.pdf]
