# Supplementary material for: Integration of mRNA and miRNA analysis reveals the molecular mechanisms of sugar beet (Beta vulgaris L.) response to salt stress
Source: Sci Rep. 2023 Dec 12;13:22074. doi: 10.1038/s41598-023-49641-w (PMC10716384; doi:10.1038/s41598-023-49641-w)
Supplement: Supplementary file 1 — Supplementary Information. [file Figture S2.pdf › Figture S2.pdf]

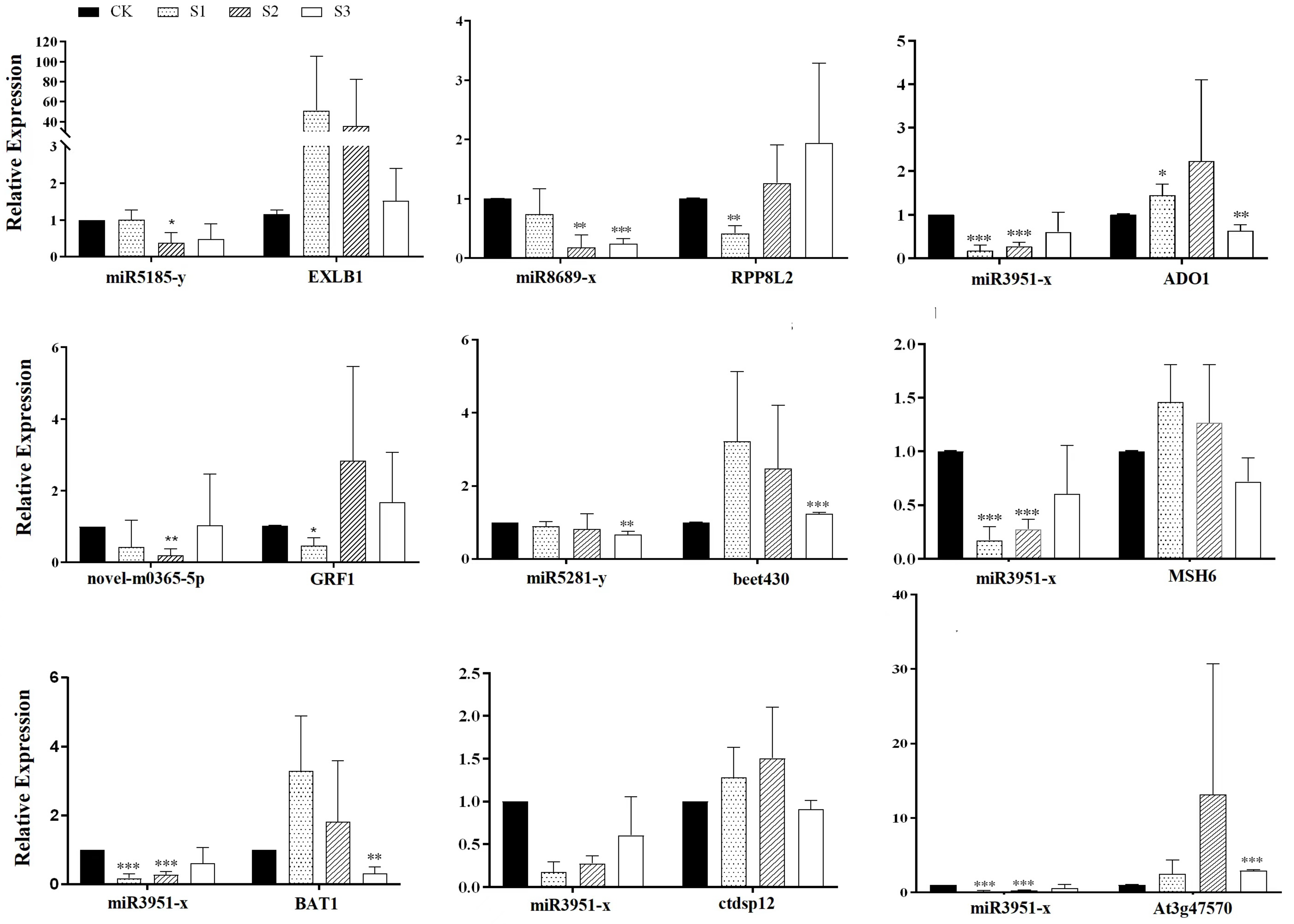

Fig. S2. Reliability of the transcriptome data was verified by qRT-PCR.
(The expression trend of mRNA and miRNA detected by qRT-PCR is opposite, proving the reliability of the results).
